# Improved Field Electron Emission Properties of Phosphorus and Nitrogen Co-Doped Nanocrystalline Diamond Films

**DOI:** 10.3390/nano10061024

**Published:** 2020-05-27

**Authors:** Fernando Lloret, Kamatchi Jothiramalingam Sankaran, Josué Millan-Barba, Derese Desta, Rozita Rouzbahani, Paulius Pobedinskas, Marina Gutierrez, Hans-Gerd Boyen, Ken Haenen

**Affiliations:** 1 Institute for Materials Research (IMO), Hasselt University, 3590 Diepenbeek, BelgiumDerese.desta@uhasselt.be (D.D.); Rozita.rouzbahani@uhasselt.be (R.R.); Paulius.pobedinskas@uhasselt.be (P.P.); hansgerd.boyen@uhasselt.be (H.-G.B.); 2 IMOMEC, IMEC vzw, 3590 Diepenbeek, Belgium; 3 Department Fisica Aplicada, Universidad de Cádiz, 11510 Puerto Real, Spain; 4 Department Ciencia de los Materiales e IM y QI, Universidad de Cádiz, 11510 Puerto Real, Spain; josmilbar@gmail.com (J.M.-B.); marina.gutierrez@uca.es (M.G.)

**Keywords:** nanocrystalline diamond, field electron emission, phosphorus, nitrogen, conductive atomic force microscopy, transmission electron microscopy

## Abstract

Nanocrystalline diamond (NCD) field emitters have attracted significant interest for vacuum microelectronics applications. This work presents an approach to enhance the field electron emission (FEE) properties of NCD films by co-doping phosphorus (P) and nitrogen (N) using microwave plasma-enhanced chemical vapor deposition. While the methane (CH_4_) and P concentrations are kept constant, the N_2_ concentration is varied from 0.2% to 2% and supplemented by H_2_. The composition of the gas mixture is tracked in situ by optical emission spectroscopy. Scanning electron microscopy, atomic force microscopy (AFM), transmission electron microscopy, and Raman spectroscopy are used to provide evidence of the changes in crystal morphology, surface roughness, microstructure, and crystalline quality of the different NCD samples. The FEE results display that the 2% N_2_ concentration sample had the best FEE properties, viz. the lowest turn-on field value of 14.3 V/µm and the highest current value of 2.7 µA at an applied field of 73.0 V/µm. Conductive AFM studies reveal that the 2% N_2_ concentration NCD sample showed more emission sites, both from the diamond grains and the grain boundaries surrounding them. While phosphorus doping increased the electrical conductivity of the diamond grains, the incorporation of N_2_ during growth facilitated the formation of nano-graphitic grain boundary phases that provide conducting pathways for the electrons, thereby improving the FEE properties for the 2% N_2_ concentrated NCD films.

## 1. Introduction

Diamond is a versatile material whose properties make it interesting for a wide range of applications, from abrasives to power electronics [1]. Among these applications, polycrystalline diamond-based field emitters have attracted significant interest in vacuum microelectronics due to their negative electron affinity for electron emission, strong bonding structure, extreme hardness to withstand ion bombardment, and good thermal and electrical conductivities to handle high currents [2,3].

Chemical vapor deposition (CVD) is the most used technique for producing polycrystalline diamond. Using methane and hydrogen as gas precursors, it is possible to obtain diamond films with grain sizes ranging from tens of nanometers to hundreds of microns. Microcrystalline diamond (MCD) films possess micron-sized grains resembling monocrystalline diamond, implying a wide bandgap and hindering the field electron emission (FEE) behavior because of a shortage of electrons in the conduction band [4]. As grain boundaries are known to be more conductive due to the presence of sp^2^-bonded carbon, efforts must be directed to a reduction of the diamond grain size. Nitrogen addition in CH_4_/H_2_ plasma effectively reduces the size of the diamond grains from micron to nanosized, yielding nanocrystalline diamond (NCD) films. It also facilitates the formation of nanographitic phases in the grain boundaries, for which an improvement in the electrical conductivity and subsequent FEE properties have been demonstrated [5,6,7,8]. The grain boundary conduction mechanism has been proposed for the enhancement of FEE properties of these N-doped NCD films [9,10]. Still, the nanosized diamond grains are insulating in nature, which limits conduction to the grain boundaries, thereby limiting the FEE properties of N-doped NCD films. On the other hand, it is known that P forms a donor in both single as well as polycrystalline diamond, with the P atom mostly incorporating in substitutional positions in the grains [11,12].

Here we present an approach to synthesize phosphorus and nitrogen co-doped NCD films, with the aim of improving the FEE properties of N-doped NCD films. With the target of obtaining a large proportion of conducting grain boundary phases (due to N-doping) between conducting P-doped diamond grains, P and N co-doped NCD films were deposited on Si substrates by microwave plasma-enhanced chemical vapor deposition (MWPECVD), and the optimum growth conditions to enhance the FEE properties were determined. The composition of the gas mixture during growth was tracked in situ by optical emission spectroscopy (OES). The resulting NCD films were characterized by scanning electron microscopy (SEM), atomic force microscopy (AFM), Raman spectroscopy, transmission electron microscopy (TEM), and conductive AFM (C-AFM).

## 2. Materials and Methods

A total of five 10 × 10 mm^2^ n-type mirror-polished (100)-oriented silicon substrates (10–20 kΩ·cm) were exposed to an O_2_ plasma for 1 min at 200 W and 50 sccm total flux in order to prepare the surface for diamond seeding. Oxygen plasma was used to clean the surface of organic contamination and to improve the hydrophilicity of the surface. Substrates were then seeded by drop-casting with a water-based colloidal suspension of ultra-dispersed detonation nanodiamond of size 6–7 nm (NanoCarbon Institute Co., Ltd, Japan.) and subsequent spin-drying [13]. Growth was carried out in an MWPECVD ASTeX reactor. A mixture of CH_4_, H_2_, N_2_, and PH_3_ gases was used with a total flux of 500 sccm. CH_4_ and PH_3_ were kept constant at 1% and 0.01%, respectively, in all the deposition experiments, while the proportion of N_2_ was varied from 0.2% to 2% and complemented by H_2_. The working pressure and microwave power were set at 70 Torr and 2100 W, respectively, leading to a temperature of the growth surface in the range of 760 to 800 °C, which was measured by a single-color optical pyrometer with the emissivity set to 0.3. An AvaSpec-2048 spectrometer with a spectral resolution of 1 nm per pixel was used to track the composition of the plasma region in contact with the sample by OES. All the samples were grown for a thickness of ~200 nm. Table 1 summarizes the growth conditions used for each sample.

Grain morphology was studied by SEM using an FEI Quanta 200 FEG operated with 15 kV accelerating voltage, while surface roughness was evaluated by AFM using a Bruker Multimode 8 in tapping mode. The crystalline quality of the films was examined using a Horiba Jobin Yvon T64000 Raman spectrometer equipped with a BXFM Olympus 9/128 microscope, in combination with a Horiba Jobin Yvon Symphony CCD detector and a 488 nm Lexell SHG laser. The deposited NCD layers were studied in planar-view preparation by TEM at 200 keV accelerating voltage using a JEOL 2100 TEM. TEM samples were prepared by mechanical polishing and ion milling.

Room-temperature field electron emission (FEE) measurements of the NCD films were carried out by a diode-type apparatus inside a vacuum chamber at a pressure of 2 × 10^−8^ mbar. An Mo rod of 3 mm diameter acted as an anode, and the NCD films were used as the cathode. A micrometer feed-through with 1 µm resolution was used to control the anode-to-cathode distance. A Keithley 6517B electrometer (Keithley Instruments, Inc., Cleveland, OH, USA) was used to acquire the current–voltage (*I–V*) characteristics. C-AFM (NX-10, Park Systems) measurements were carried out at a negative substrate bias voltage of −5 V using an Au-coated silicon cantilever (NPG-10) from Bruker.

## 3. Results and Discussion

**Figure 1:**
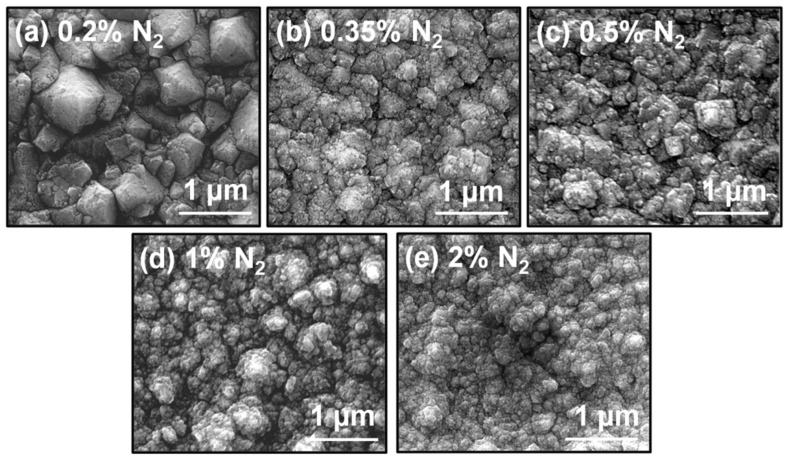
SEM micrographs of the samples grown with (**a**) 0.2% N_2_, (**b**) 0.35% N_2_, (**c**) 0.5% N_2_, (**d**) 1% N_2_ and (**e**) 2% N_2_.

SEM observations were carried out in order to analyze the changes in the grain morphology of the NCD films. The SEM micrograph of the 0.2% N_2_ NCD sample (Figure 1a) showed a larger grain size of 1 µm diameter for the biggest grains. Most of the grains looked (111)-faceted, with some not very evident re-nucleation in the edges. Figure 1b shows the surface of the sample growth at 0.35% N_2_, where the effect of the increase of nitrogen on the grain morphology is clear. Grains were drastically reduced to nanometric scale, where the largest ones were only a few hundred nanometers in diameter. Facets were not as evident in this case. The grain size continued reducing, and the shape became so-called cauliflower-like when nitrogen content was increased. The NCD sample grown at 2% N_2_, shown in Figure 1e, clearly exhibited small grains with many different facets in a clear cauliflower morphology that predicted larger grain boundary regions.

**Figure 2:**
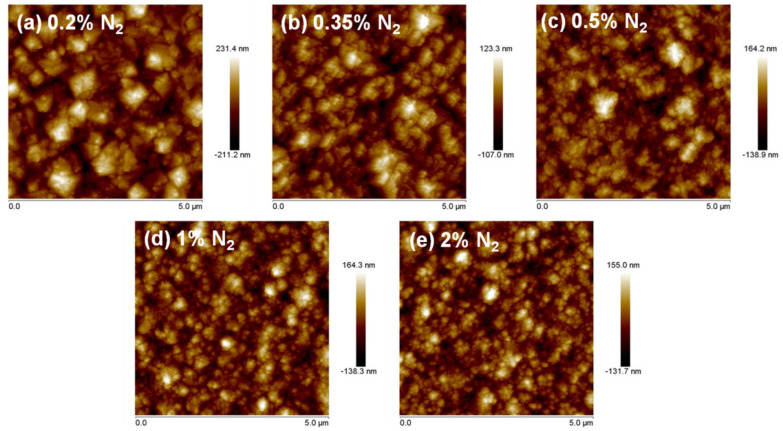
Topographic AFM images of the samples grown with (**a**) 0.2% N_2_, (**b**) 0.35% N_2_, (**c**) 0.5% N_2_, (**d**) 1% N_2_ and (**e**) 2% N_2_.

Figure 2 shows AFM micrographs of each sample. The average roughness reduced with increasing nitrogen content, resulting in the *Ra* values of: 67, 40, 34, 32, and 30 nm for 0.2%, 0.35%, 0.5%, 1%, and 2% N_2_ content, respectively. Thus, from 0.2% to 0.5%, there was a huge reduction of roughness, indicating a big change in the morphology of the diamond grains. The surface remained smoother at higher nitrogen concentration, but variations were not as abrupt. These results were in good agreement with the SEM micrographs (cf. Figure 1).

**Figure 3:**
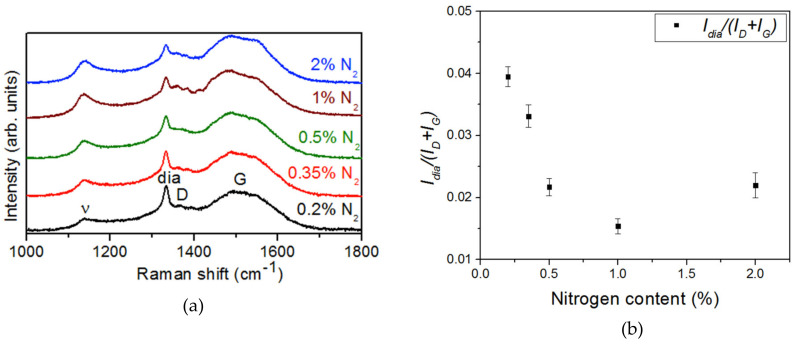
(**a**) Raman spectra of the NCD samples grown with different N_2_ concentrations. (**b**) *Dia* and G + D bands ratio (*I_dia_/(Id + Ig)*) for each nitrogen content.

Figure 3 shows the Raman spectra of these samples. This technique allowed the evaluation of the crystalline quality of the NCD layer by exploring the bonding character of the carbon phases present in the samples. The two peaks located at about ~1350 cm^−1^ (D-band) and ~1580 cm^−1^ (G-band) are characteristic of carbon materials, and correspond to breathing and stretching modes in sp^2^-bonded carbon clusters, respectively [14,15]. Moreover, a third characteristic peak (labeled *“dia”*) at ~1332 cm^−1^ was observed that relates to sp^3^ diamond bonds. This peak looked sharp due to the microcrystalline character of the diamond film and ensured the diamond phase of the deposited film. The sharpness of the *“dia”* peak was related to defects and/or stress in the diamond phase and could be estimated by its FWHM. After Lorentz deconvolution, results of 14.0 ± 0.5, 15.7 ± 0.7, 12.8 ± 0.8, 20.3 ± 1.0, and 16.2 ± 1.4 cm^−1^ were obtained for 0.2%, 0.35%, 0.5%, 1%, and 2% nitrogen, respectively. There was no straightforward correlation between the FWHM and the nitrogen content. Consequently, there was no evidence of any effect on the stress or crystalline quality due to the addition of nitrogen to the gas mixture during growth. Nevertheless, this peak reveals that the diamond phase remained in all samples.

A fourth peak appeared in the *v* band at ~1140 cm^−1^, which is the specific mode corresponding to *trans*-polyacetylene (*t*-PA) segments and the vibration of sp^2^ carbon atoms bonded at the grain boundaries [16]. A second *v* band was at ~1460 cm^−1^ and overlapped the G band. Raman spectra showed the increase of the *v* band with N_2_ concentration, which resulted in the formation of nanosized diamond grains (in concordance with SEM micrographs (cf. Figure 1) and, consequently, more sp^2^ carbon bonded grain boundaries [8].

The sp^2^/sp^3^ ratio was estimated by the comparison of *“dia”*, D, and G bands using the *I_dia_/(Id + Ig)* ratio. Figure 3b shows this ratio, after deconvolution, as a function of the nitrogen concentration. There was an increase of the sp^2^ phases with the increase of nitrogen in the gas phase that tended to stabilize at 1%.

**Figure 4:**
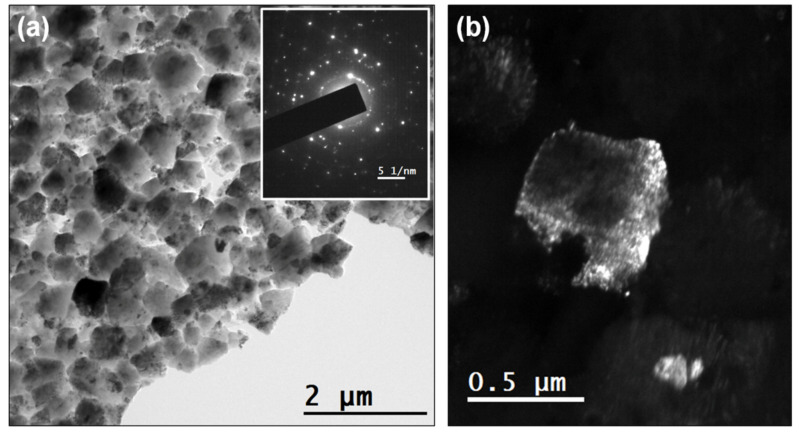
(**a**) Bright field (BF) TEM micrograph of sample grown with 0.2% N_2_. The inset of (a) shows the SAED pattern using an aperture with 1 µm diameter. (**b**) Dark field (DF) micrograph of one reflection that shows one of the grains with good contrast.

**Figure 5:**
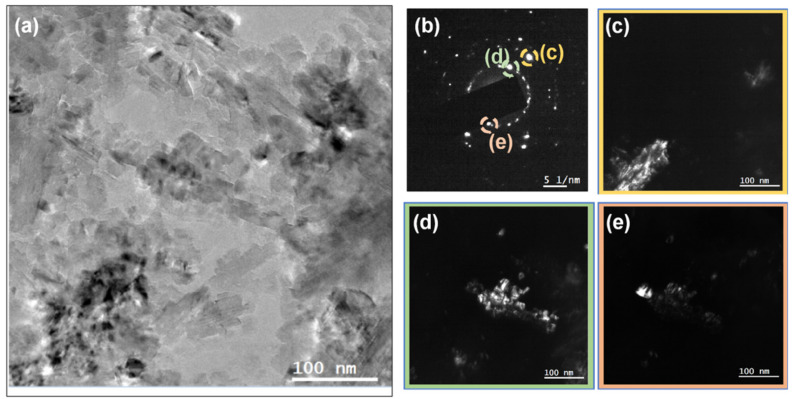
(**a**) BF TEM micrograph of the sample grown with 2% N_2_. (b) SAED pattern of the region shown in (**a**) using an aperture with 200 nm diameter. (**c**,**d**,**e**) DF micrographs of the reflections marked on the SAED pattern.

The microstructure of the NCD samples was examined using TEM. Figure 4a shows a bright field (BF) TEM micrograph of the 0.2% N_2_ NCD sample. The selected area electron diffraction (SAED) pattern of an area 1 µm in diameter is shown as an inset of Figure 4a. Bigger grains that were hundreds of nanometers in size are evidenced, one of which is shown in the dark field (DF) mode in Figure 4b. The sample grown with 2% nitrogen is shown in BF mode in Figure 5a. The smaller grains required higher magnification. SAED was performed in the area with an aperture of 200 nm diameter (Figure 5b). Three different reflections in DF mode are provided in Figure 5c–e to show the grains that were tens of nanometers in size.

**Figure 6:**
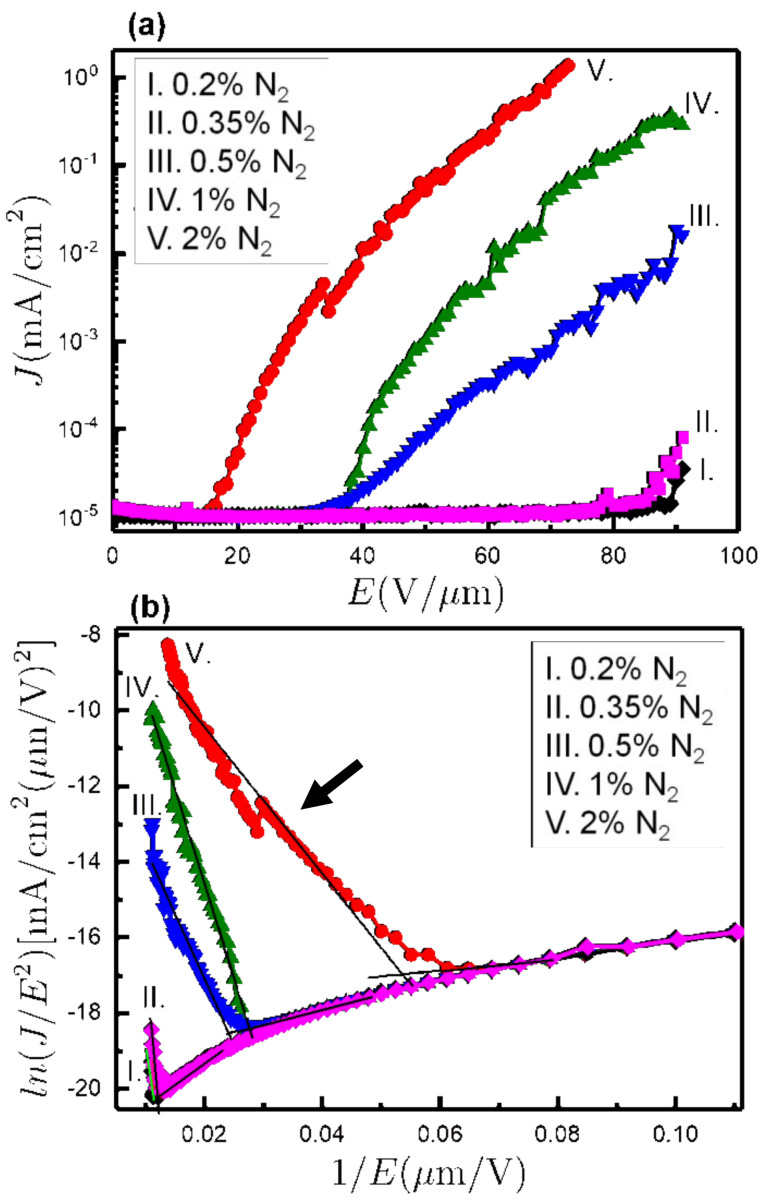
(**a**) FEE current density (*J*) as a function of applied field (*E*). (**b**) Fowler–Nordheim (FN) plots of the corresponding *J*–*E* characteristic curves. All the results are shown for the five samples grown at different N_2_ contents.

Figure 6a shows the FEE current density (*J*) versus applied field (*E*) of all co-doped NCD samples. The FEE results were Fowler–Nordheim (FN) modeled [8], from which the turn-on field (*E*_0_) was determined for each film, as shown in Figure 6b. These values, summarized in Table 2, show a drastic reduction of *E*_0_ for higher N_2_ content combined with a big increase in the current density. In this way, for the 0.2% N_2_ sample, the turn-on field was 83.7 V/µm, and the current density at 53 V/µm applied field was 10^−5^ mA/cm^2^, respectively. Similar values were obtained for 0.35% N_2_ (*E*_0_ = 88.5 V/µm and *J* = 10^−5^ mA/cm^2^) even if the SEM micrographs showed clear differences in the grain morphology between the 0.2% N_2_ and 0.35% N_2_ NCD samples (cf. Figure 1). Nevertheless, at an N_2_ ratio of 0.5%, the electric field for which the film can be turned on was reduced to half (i.e., *E*_0_ = 41.0 V/µm), while the current density increased to *J* = 22 × 10^−5^ mA/cm^2^. Interestingly, for the 2% N_2_ sample, *E*_0_ reached its minimum value of 18.7 V/µm, with the current density reaching 13 × 10^−2^ mA/cm^2^. Moreover, the *β* values were estimated from the slope (*m*) of FN plots (see the arrow in Figure 6b) using the relation

*β* = [− 6.8 × 10^3^*φ*^3/2^]/*m*

where *φ* is the work function, and *m* is the slope of the FN plot. The calculated *β* values were 27, 12, 130, 89, and 230 for 0.2%, 0.35%, 0.5%, 1%, and 2% N_2_ content, by taking the *φ* value as 5.0 eV, respectively [17]. The nanosized diamond grains with wider grain boundaries in the 2% N_2_ NCD sample were the main factors for the larger *β* value. Consequently, the 2% N_2_ sample showed better FEE characteristics, viz. lower *E*_0_ value, higher *J* value, and larger *β* values as compared with the other co-doped samples in this study.

**Figure 7:**
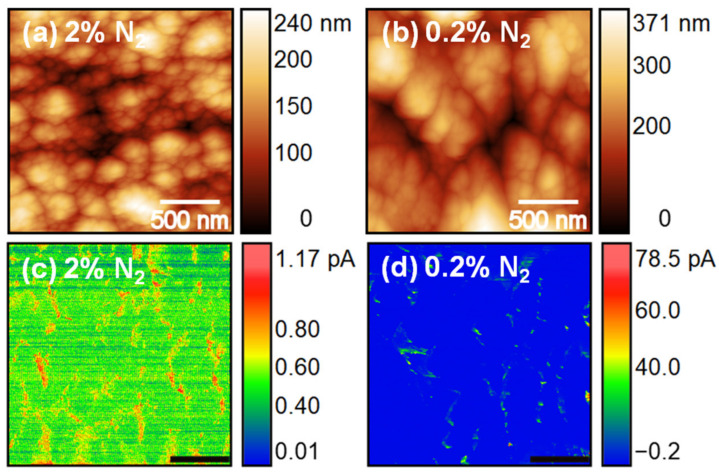
C-AFM topographic images (**a**,**b**) and the corresponding current signal (**c**,**d**) of 2% N_2_ NCD and 0.2% N_2_ NCD samples, respectively.

To determine the reason for the enhancement of the FEE characteristics of the 2% N_2_ NCD sample, C-AFM measurements were carried out to detect the current conduction paths of the NCD films. Figure 7a,c shows the C-AFM topographic image and the corresponding current signal for 2% N_2_ NCD. C-AFM measurements of the 0.2% N_2_ NCD sample were also carried out for comparison (Figure 7b,d). The brighter contrast in the current images signifies a larger emission current. For both samples, larger emission current was observed from the grain boundaries, which was due to the presence of sp^2^-bonded carbon phases at the grain boundaries confirmed from the Raman results (Figure 3). Harniman et al. [18] also observed that FEE from diamond surfaces originates preferentially from the grain boundaries and not from other topographical features. This was observed in low-doping samples where grain boundaries remained relatively conductive compared to the bulk grains. In these samples, the electrons used the grain boundaries as a path to climb from the base of the film to the surface, where they were emitted. The situation changed when the grains were conductive, causing some of the current to be transported through the grains themselves and be emitted from the facets. In our results, the number density of highly conductive regions was larger for 2% N_2_ NCD than that of 0.2% N_2_ NCD because 2% N_2_ NCD films contained more sp^2^-bonded phases at the grain boundaries, which was in accordance with the Raman studies. Moreover, emission sites were also observed from the P-doped diamond grains of 2% N_2_ NCD. Hence the C-AFM measurements clearly illustrate the existence of a high density of local electron emission sites in 2% N_2_ NCD, implying the formation of conductive nanochannels at the grains and grain boundaries resulting in the enhanced FEE properties [19]. In our previous studies, it was observed that phosphorus was doped directly inside the lattice of the diamond [11], which made the diamond grains conductive. However, the main factor responsible for the reduction of nanosized diamond grains and the induction of sp^2^-bonded carbon phases in the grain boundaries is still not clear.

**Figure 8:**
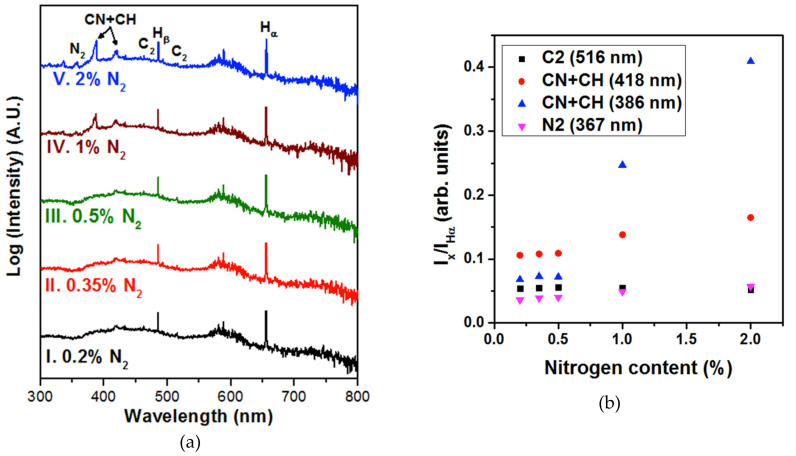
(**a**) OES spectra recorded during the deposition process of the five samples labelled by their N_2_ amount. (**b**) Intensity of C_2_, N_2_, and CN + CH peaks normalized to the H_α_ peak for each nitrogen content used.

To determine the factors responsible, we carried out in situ OES measurements during the sample growth. Figure 8a shows optical emission spectra taken from the plasma during NCD sample growth from CH_4_/H_2_/N_2_/PH_3_ gas mixtures with varying N_2_ concentrations. The H_α_ and H_β_ lines are the Balmer atomic hydrogen emission lines observed at ~655 and ~484 nm [20,21], respectively, whereas the CN violet system (386.3 and 418.1 nm) and the N_2_ line at 357.3 nm [22] were induced due to the addition of N_2_. The intensity of CN and N_2_ lines increased as the N_2_ concentration in the plasma increased, and reached a maximum for 2% N_2_ NCD (Figure 8, spectrum V). Generally, the CH_4_ species in the plasma dissociated and formed CH_x_ species (x = 1, 2, 3). However, the CH lines were overlapped by the CN line [23,24], and lines at 386.3 and 418.1 nm were marked as CN + CH. The C_2_ swan system at 516.0 nm was also observed with low intensity [25]. Our previous studies stated that the presence of CH and C_2_ species are responsible for the origin of nanosized diamond grains because the C_2_ species re-nucleate diamond easily, whereas the CH species passivate the diamond nuclei and efficiently result in the formation of nanosized diamond grains [10,25]. Based on the recorded spectra, Figure 8b shows a comparative graph of the species in the plasma during the growth. At a high concentration of N_2_ (2%) in the plasma, the CN + CH peaks increased. This was probably due to an increase of the CN species that were more present as compared to the CH species [8]. Moreover, the NCD films were grown at a high growth temperature (>700 °C), which made it energetically favorable for the CN species to be more active, likely resulting in the formation of sp^2^-bonded carbon at the grain boundaries [10,26]. Hence CN, CH, and C_2_ species were the main factors for the origin of the nanosized diamond grains and the induction of sp^2^-bonded carbon phases in the grain boundaries of 2% N_2_ NCD films.

## 4. Conclusions

From this study, the combined effect of PH_3_ and N_2_ on the drastic reduction of diamond grain size and the consequent enlarging of the grain boundaries was clearly evidenced. Roughness reduction was illustrated by AFM, while the reduction of grain size was pointed out by SEM and TEM. C-AFM studies showed that more emission sites from diamond grains and sp^2^-bonded carbon grain boundaries of an NCD sample co-doped with P and N, with 2% N_2_ used in the gas phase, resulting in enhanced FEE properties, viz. a low *E*_0_ value of 18.7 V/µm, a high current density of 13 × 10^−2^ mA/cm^2^, and a field enhancement factor of 230. The CN, CH, and C_2_ species in the growth plasma were considered the prime factors for the origin of the nanosized diamond grains and the induction of sp^2^-bonded carbon phases in the grain boundaries of the co-doped NCD films.

## Figures and Tables

**Table 1:** Growth conditions used during the microwave plasma-enhanced chemical vapor deposition (MWPECVD) deposition of the nanocrystalline diamond (NCD) layers, Ra determined by AFM and full width at half maximum (FWHM) of the *dia* peak, corresponding to the Raman spectrum.

Power	Pressure	Temperature	CH_4_	PH_3_	N_2_	Ra	FWHM *“dia”*
2100 W	70 Torr	760–800 °C	1%	250 sccm	0.2%	67 nm	14.0 cm^−1^
2100 W	70 Torr	760–800 °C	1%	250 sccm	0.35%	40 nm	15.7 cm^−1^
2100 W	70 Torr	760–800 °C	1%	250 sccm	0.5%	34 nm	12.8 cm^−1^
2100 W	70 Torr	760–800 °C	1%	250 sccm	1%	32 nm	20.3 cm^−1^
2100 W	70 Torr	760–800 °C	1%	250 sccm	2%	30 nm	16.2 cm^−1^

**Table 2:** Field electron emission (FEE) properties of P and N co-doped NCD films with varying N_2_ concentrations.

N_2_	*E*_0_ (V/µm)	*β*	*J* at 53 V/µm
0.2%	83.7	27	1 × 10^−5^ (mA/cm^2^)
0.35%	88.5	12	1 × 10^−5^ (mA/cm^2^)
0.5%	41.0	130	22 × ^10−5^ (mA/cm^2^)
1%	36.2	89	26 × 10^−4^ (mA/cm^2^)
2%	18.7	230	13 × 10^−2^ (mA/cm^2^)
